# Social Cognition and Friendships in Adolescents With Autistic-Like Experiences and Psychotic-Like Experiences

**DOI:** 10.3389/fpsyt.2020.589824

**Published:** 2021-01-15

**Authors:** Hester Sijtsma, Nikki C. Lee, Miriam Hollarek, Reubs J. Walsh, Mariët van Buuren, Barbara R. Braams, Lydia Krabbendam

**Affiliations:** Section of Clinical Developmental Psychology, Faculty of Behavioral and Movement Sciences, Research Institute LEARN!, Institute of Brain and Behavior, Vrije Universiteit Amsterdam, Amsterdam, Netherlands

**Keywords:** autistic-like experiences, psychotic-like experiences, social cognition, friendships, non-clinical samples

## Abstract

Autism spectrum conditions (ASC) and schizophrenia spectrum conditions (SSC) are both characterized by changes in social-cognitive functioning. Less is known about the overlap and the differences in social-cognitive functioning when comparing individuals with subclinical levels of ASC and SSC, while studies in non-clinical samples have the benefit of avoiding confounds that are present in clinical groups. Therefore, we first examined how autistic-like experiences, positive psychotic-like experiences and the co-occurrence of both correlated with the performance on an extensive battery of social cognition tasks in young adolescents. Second, we examined the effect of autistic-like experiences, psychotic-like experiences and their co-occurrence on friendships in daily life. A total of 305 adolescents (*M*age = 12.6, *sd* = 0.4, 147 boys) participated in the current study. A battery of social cognition tasks, comprising the Reading the Mind in the Eyes task, Dot perspective task and trust game were individually administered in a classroom setting, along with a friendship peer nomination questionnaire. Results indicated no evidence for a relationship between the performance on the social cognition battery and subclinical experiences of autism and/or psychosis. However, results did show that the amount of autistic-like experiences of adolescents were associated with being less often selected as a friend by their peers. By contrast, no relationship between self-reported friendships and autistic-like experiences was found. Neither a relationship between friendships and psychotic-like experiences was reported. This study provides initial evidence that information provided by peers may shed light on (altered) social behavior associated with autistic-like experiences that is not apparent on performance measures, as well as elucidate possible differences between autistic- and psychotic-like experiences.

## Introduction

Both autism spectrum conditions (ASC) and schizophrenia spectrum conditions (SSC) are neurocognitive conditions characterized by altered social-cognitive functioning. Since their early conception, much attention has been paid to describing the overlap and differences between ASC and SSC (1, 2). Both conditions and their subclinical expressions are related to a decrease in social or emotional understanding, difficulties during social interactions and problems in interpersonal relationships (3–6). Research in non-clinical populations may help to further elucidate the nature of difficulties in social functioning and the relationship between the two conditions (7). Adolescence is a particularly interesting period for this research, as this phase is characterized by extensive changes in social cognition (8) and by significant increases in the prevalence of psychopathology (9, 10). Furthermore, altered social functioning in non-clinical samples may impact peer relations, which typically increase in quantity and in meaning during adolescence (11). Therefore, the first aim of the current study was to examine the overlap and differences between autistic-like experiences and psychotic-like experiences on a battery of social cognition tasks in young adolescents. Secondly, we tested how subclinical experiences impact adolescent friendships within the classroom. The study was conducted in a non-clinical sample, which avoids confounds that may arise in clinical groups, for example use of medication, differences in attention and motivation which can impact the performance on cognitive tasks, and the presence of comorbidities or interacting cognitive difficulties (7).

Studies on social cognition in clinical samples have demonstrated altered Theory of Mind (ToM) abilities in both ASC (12, 13) and SSC (14). Theory of mind is the ability to understand that people have their own mental states, such as thoughts and desires, and that these affect behavior (13). There is evidence for other social-cognitive dysfunctions as well. A recent meta-analysis revealed similarly decreased performance between ASC and SSC on measures of emotion recognition, emotional intelligence, social skills and ToM abilities (15). The only differences between the groups were found on tasks of facial emotion perception, where those with SSC outperformed those with ASC. A number of studies have examined the social-cognitive changes associated with either ASC or SSC phenotypes in non-clinical groups (16–20). Most findings support the suggestion that those with subclinical experiences have similar (though milder) difficulties in social interactions to those who have been diagnosed with either condition. Thus, poorer ToM, emotion recognition and social skills have been associated with higher levels of autistic-like experiences (17, 20–22) and psychotic-like experiences have been associated with decreased ToM performance in adolescents and adults (16, 18, 19, 23). According to a recent meta-analysis ToM difficulties are related to both positive and negative psychotic-like experiences (24).

Evidence from non-clinical samples suggests that childhood autistic-like experiences are associated with psychotic-like experiences during adolescence [(25, 26), mean age of both studies was 12 years]. Little is known about the co-occurrence of autistic-like experiences and positive psychotic-like experiences in relation to social cognition. In a previous study, perspective-taking skills in a non-clinical young adult sample (mean age 21) were examined (27). Results showed that having either autistic-like experiences or psychotic-like experiences was associated with increased perspective-taking errors, but this effect was reduced in the group with a combination of both high autistic tendencies and high psychosis proneness. The performance of the latter group was similar to that of those with low autistic tendencies and low psychosis proneness (27). These findings provide initial support for the diametric model, which suggests that the relationship between ASC and SSC can be viewed as that ASC and SSC are on opposite extremes of a social-cognition continuum, in which social-cognitive skills are underdeveloped in ASC and overdeveloped in SSC (2, 28). According to the diametric model, having both subclinical symptoms of ASC and SSC may result in typical levels of performance as these may have an ameliorating effect on one another (2, 28). These results emphasize the importance of examining the co-occurrence of ASC and SSC symptoms.

It has been suggested that differences in social and communication skills between those with and without autistic-like experiences make it more challenging to foster and maintain friendships (5). Studies have shown that having autistic-like experiences is related to a poorer quality of friendships and to fewer friendships (5, 6). Having psychotic-like experiences that consist of unusual beliefs about other people (such as being persecuted) can make someone reluctant to socialize with others (3). A study has shown that having psychotic-like experiences is associated with having problems in peer relationships (3). Early adolescence is a critical period for social development, during which peers become increasingly important and friendships are formed (29). As adolescents spend a substantial amount of their time at school, the classroom is an important social environment for the formation of friendships. These social relationships can be examined using social network analysis in which both the adolescent and their classmates report on their friendships. This method accounts for the adolescent's own view on their friendships, but also asks their peers to report these relationships. As mentioned by Wainer et al. (6), relying solely on self-report data when examining associations between subclinical experiences and friendships may result in biased results (commonly referred to as common-method variance), and therefore peer report data may add valuable, and possibly new, insights. Social network analysis provides a measure that indicates the number of peers a person selects as friends (outgoing ties: one's own perspective) and how many peers select the person as a friend (incoming ties: peer's perspective).

In the current study, 305 young adolescents were tested on a battery of social cognition tasks combined with measures of their social relationships within their classroom. First, we examined how young adolescents with autistic-like experiences, with positive psychotic-like experiences and with the co-occurrence of both perform on social cognition tasks, specifically visual perspective taking (the Dot perspective task), mental state recognition (the Reading the Mind in the Eyes task), and interpersonal trust (the trust game). We expected a negative association between autistic-like experiences and performance on social cognition tasks and between psychotic-like experiences and performance on social cognition tasks. Based on the results of the study of Abu-Akel et al. (27), we investigated if the co-occurrence is related to better performance on social cognition tasks compared to high levels of either autistic-like experiences or psychotic-like experiences, as the combined traits would have an ameliorating effect on one another, supporting the diametric model. Second, we examined the effect of autistic-like experiences, psychotic-like experiences, and the co-occurrence of both on friendships in the classroom setting. For both autistic-like experiences and psychotic-like experiences we hypothesized a negative association with the number of friendships (for both outgoing ties and incoming ties).

## Methods

### Participants

Participants in the current study were taken from wave 1 and wave 2 of the second cohort of the longitudinal #SOCONNeCT project. A total of 647 adolescents provided written informed consent before the start of wave 1 of the #SOCONNeCT project. Data collection within the #SOCONNeCT project consisted of six waves, twice per school year, starting in the first year of high school. Participants were recruited from eight high schools across The Netherlands. All participants were enrolled in the senior general secondary educational track or a pre-university educational track, which constitute the higher tracks within the Dutch education system (top 40% of pupils based on academic achievement). Participants from the larger sample were included in the analyses of the current study if they were in classes where a minimum of 70% of pupils participated and had complete data on all relevant variables, that is the Diagnostic Interview for Children (DISC-C), the Autism Symptom SElf-ReporT for adolescents and adults (ASSERT), the Reading the Mind in the Eyes task (RMET), the Dot perspective task, the trust game, the Raven's Progressive Matrices (SPM), and the urbanization and SES measures. A minimum of 70% participants within a class is advised to create reliable social network positions, although this cut-off is under continuous discussion (30, 31). From a total of 33 classes, 15 met the 70% cut-off rate. Based on these criteria 305 participants (*M*age = 12.6, *sd* = 0.4, 147 boys) were included in the final sample. Schools received €7.50 per participating pupil per wave to use for a class activity. The #SOCONNeCT project was approved by the Scientific and Ethical Review Board of the Faculty of Behavioral and Movement Sciences of the Vrije Universiteit Amsterdam.

### Procedure

Both the participant and the parent(s)/caregiver(s) of the participant gave active informed consent (either via e-mail or a printed version) for participation in the #SOCONNeCT project. Pupils and parents were contacted via schools and informed about the project with a letter and an information evening in which the aims and the procedure of the project were explained. To make sure the participants understood the protocol of the study and the research aims, data collection started with an extensive explanation of what research entails and on the rights that participants have. Every participant then signed an informed consent.

Data collection was done at school under supervision of researchers and trained research assistants and lasted about 90 min, including classroom explanations and the administration of tasks and questionnaires not analyzed in the current study. To make sure participants understood the questionnaires and tasks, the explanation was adjusted according to feedback received by several focus groups of adolescents. Also, the researchers covered frequently asked questions that were created after discussion with the focus groups (these questions were mostly about what research is, and whether the research was part of the education curriculum of their school). Furthermore, for the trust game a joint, extensive explanation was given beforehand. Then, on a laptop, each participant individually had to answer multiple questions about the method of the game correctly before the participant was able to start the game. The Dot perspective task started with 10 practice rounds (five per block) and these were repeated until the correct answer was given. Afterwards, a researcher or research assistant went by every participant individually to make sure the method of the game was clear and only then, the participant was able to start the task. Furthermore, throughout data collection participants were given the opportunity to ask questions to the researchers at all times. Each participant completed the tasks and questionnaires individually on a laptop and on an iPad provided by the researchers. All materials that were administered on laptops were tested and validated on laptops during the focus groups and the same was true for the materials that were administered on iPads. The RMET, the Dot perspective task, the trust game, the peer nomination questionnaire and the urbanization and SES measures were administered in wave 1 and the DISC-C, the ASSERT and the SPM were administered in wave 2. Wave 1 and wave 2 were administered within the same school year (~6 months apart).

### Materials

#### Diagnostic Interview for Children (DISC-C)

Four items from the self-report DISC-C (schizophrenia section) (32) were used to assess subclinical positive psychotic-like experiences which could be answered with “yes” or “no”: (1) “Some people believe in mind reading or being psychic. Have other people ever read your mind?”, (2) “Have you ever had messages sent just to you through television or radio?”, (3) “Have you ever thought that people are following you or spying on you?”, (4) “Have you ever heard voices people cannot hear?” (33). The sum score on the DISC-C was used as an indication of psychotic-like experiences (range 0–4). The items were administered in Dutch (34) using an iPad. A longitudinal study showed that the four items at age 11 predicted the presence of adult psychosis at age 26 (33).

#### Autism Symptom SElf-ReporT for Adolescents and Adults (ASSERT)

The self-report ASSERT questionnaire was used to assess subclinical autistic-like experiences (35). The ASSERT consists of seven items on a three point scale (“not true”, “somewhat true”, and “certainly true”): (1) “Do you find it difficult to socialize with, or get in touch with people, especially people of your own age?”, (2) “Do you prefer to be alone rather than being together with other people?”, (3) “Do you have difficulties perceiving social cues?”, (4) “Do other people tell you that your behavior or your emotional responses are inappropriate or hurtful?”, (5) “Do you have a strong interest or hobby that absorbs so much of your time that it hampers other activities?”, (6) “Do you or do other people feel that you have very set routines or that you are very immersed in your own interests?”, (7) “Do you or do other people feel that you impose your routines or interests on others?”. The sum score on the ASSERT was used as indication of autistic-like experiences (range 0–14). A previous study found that the validity of the ASSERT as a screening instrument for the diagnosis ASC in adolescents was good (sensitivity of 0.80 and specificity of 0.86 for scores ≥8) (35). The items were translated to Dutch by a bilingual native Dutch/English speaker (including back translations and discussion of possible uncertainties) and administered using an iPad.

#### Reading the Mind in the Eyes Task (RMET, Child Version)

The child version of the RMET questionnaire consisted of 28 pictures and was used to examine mental state recognition (36). Each picture displays human eyes surrounded by four words describing mental states. The participant was asked to choose which word best described the expression of the human eyes. The items were translated to Dutch by a bilingual native Dutch/English speaker (including back translations and discussion of possible uncertainties). To analyze the effect of autistic-like experiences and psychotic-like experiences on mental state recognition skills, a sum score of correct trials on the RMET questionnaire was calculated. The RMET was administered on an iPad and took 10 min to complete. A previous study found that the validity and reliability of the RMET was good (37).

#### Dot Perspective Task

The Dot perspective task (38) was used to measure perspective taking skills and consisted of two blocks (the arrow block and the avatar block) which were administered in counterbalanced order. The arrow block served as a control condition but was not relevant to the current analyses so it will not be described here. A trial went as follows. The participant saw a room with an avatar in the middle. Dots were shown on the walls in front of and/or behind the avatar. A trial started with a fixation cross (750 ms) and this was followed by a screen that showed the word “you” or “he/she” (750 ms). The word was replaced by a digit between one and three and, after that, the room with the avatar was shown. The word “you” indicated that one had to adopt his/her own perspective to judge whether the digit corresponds to the total number of dots one sees on all of the walls. The word “he/she” meant that the participant had to adopt the perspective of the avatar to indicate whether the digit corresponds to the number of dots that the avatar is able to see (so only the number of dots on the wall that the avatar is facing). In case of correspondence between the digit and the number of dots, the participant pushed a green button on the keyboard of the laptop. In case the digit and the number of dots did not correspond, the participant pushed a red button. The participant was told to respond as quickly as possible and had a maximum of 2 s to do so. In case the participant did not respond on time, it was counted as an incorrect response. The avatar block consisted of four different trials: self-consistent trials, self-inconsistent trials, other-consistent trials, and other-inconsistent trials. In the self-consistent trial and in the other-consistent trial, the number of dots that the participant sees and the avatar is facing are the same. In the self-consistent trial the participant should take his/her own perspective and judge whether the digit and the dots on the walls correspond. Conversely, in other-consistent trials, the participant is asked to adopt the perspective of the avatar and judge whether the digit and the number of dots correspond. In the self-inconsistent trial and the other-inconsistent trial, the number of dots that the participant sees and the avatar is facing are different (because there are also dots on the walls behind the avatar) which could cause interference of one's own perspective and the perspective of the avatar. Similarly as in the consistent trials, the participant should take their own perspective in self-inconsistent trials and the avatar's perspective in the other-inconsistent trials. Within the avatar block there are 12 trials of each category of trial (so 48 trials in total) and they appear intermixed within the block. Taking into account the age of our sample, we have, compared to the original version of the Dot perspective task by Santiesteban et al. (38), shortened the number of trials based on a study by Surtees and Apperly (39). Five practice trials were played before each block and these were repeated until the correct answer was given. The Dot perspective task was administered on a laptop and took ~10–15 min to complete.

To analyze the effect of autistic-like experiences and psychotic-like experiences on perspective-taking skills, two different measures of perspective-taking were calculated and used as dependent variables. First, a measure called the “altercentric intrusion rate” was calculated by dividing the reaction time of the response in the correctly answered self-inconsistent trials by the reaction time of the response in the correctly answered self-consistent trials (40). Values >1 indicate that the avatar's perspective interfered with the participant's judgement when they had to adopt their own perspective. A second measure was called the “egocentric intrusion rate” and was calculated by dividing the reaction time of the response in the correctly answered other-inconsistent trials by the reaction time of the response in the correctly answered other-consistent trials (40). Values >1 indicated that the participant's perspective interfered with their judgement when they needed to adopt the avatar's perspective.

#### Trust Game

The multi-round trust game was used to measure trust behavior in a dynamic, simulated social interaction (41). Two conditions of a multi-round trust game were administered in counterbalanced order. The multi-round trust game is a simulated repeated social interaction in which a trustor and a trustee share money on the basis of trust. Both conditions consisted of 15 trials. A trial starts with the trustor, the participant, sharing an amount of money between 0 and 10 euros with the trustee (the partner). The invested amount is multiplied by three and received by the trustee. Next the trustee decides how much money to keep and how much money to return to the trustor. This outcome is shown to the trustor, after which the trial ends and a new trial starts. The behavior of the trustee was modeled by a computer algorithm and the trustee's return was determined by the trustor's investment multiplied by a factor (explained below). Participants were informed they were playing with an avatar, as opposed to a human partner, as we did not want to use deception.

The computer algorithm was programmed such that the trustee's behavior was equally trustworthy in the beginning of both conditions and that the trustee's behavior changed after the first five trials. From the sixth trial onwards, the behavior of the trustee in the untrustworthy condition was modeled to be untrustworthy and the behavior of the trustee in the trustworthy condition was modeled as trustworthy. The algorithms were programmed as follows. As mentioned, the trustee's return was determined by the trustor's investment multiplied by predefined factor. For the first five trials in both conditions the factor was randomly selected between 1.2 and 1.4 (in steps of 0.1). The minimum and maximum value that the factor could reach in the first five trials was 1.2 and 1.4. The factor for the second up until the fifth trial in both conditions increased with 0.1 when the trustor's investment increased compared to the investment of the previous trial. The factor stayed the same when the investment decreased or when it did not change compared to the previous trial. Then, the factor for the sixth trial in the trustworthy condition was randomly chosen between 1.5 and 2.0 (in steps of 0.1). The minimum value of the factor became 1.5 and the maximum value became 2.0. For the seventh trial up to the fifteenth trial in the trustworthy condition, the factor increased by 0.1 when the trustor's investment increased compared to the investment of the previous trial. When the trustor's investment decreased or stayed the same compared to the previous investment, the factor did not change. So, the trustee's return was always more than the trustor's investment meaning that the behavior of the trustee was trustworthy. In the untrustworthy condition, the factor for the sixth trial was randomly chosen between 0.7 and 1.3 (in steps of 0.1). The minimum value of the factor became 0.7 and the maximum value became 1.3. The factor for the seventh to the fifteenth trial decreased by 0.1 when the trustor's investment increased compared to the previous investment. The factor stayed the same when the trustor's investment decreased or when it did not change. This setup means that the trustee becomes more untrustworthy, specifically when the trustor shows trust behavior. The trust game was administered on a laptop and took 10–15 min to complete.

To analyze the effect of autistic-like experiences and psychotic-like experiences on trust behavior we used three measures of trust behavior namely baseline trust (the mean of the first trial investment in the trustworthy condition and the first trial investment in the untrustworthy condition) and average trust behavior in both conditions (the average investment of all trials separately for the two conditions).

#### Friendship Measures

A peer nomination questionnaire was used to measure social relationships in daily life. For this study, the question “Who are your friends in your class?” was used. All participants within a class answered this question, which provided us information on the dynamics of a complete social network. The names of all participating pupils in the classroom were listed on an iPad screen and participants could select a maximum of 15 friends. The indegree measure was based on the sum of incoming friendship nominations (that is, the number of pupils selecting a participant as a friend). The outdegree measure was based on the sum of outgoing friendship nominations (that is, the number of pupils a participant selects as friends). Additional peer nomination questions were administered as part of the #SOCONNeCT project, but were not used in the current analyses. The peer nomination questionnaire was administered on an iPad and took 5–10 min to complete.

#### The Raven's Standard Progressive Matrices Test (SPM)

The SPM was administered to assess non-verbal intelligence (42). The SPM consists of five sets covering 60 analogy problems. Each problem displayed an array of pictures with one picture missing. The order of the pictures was based on a rule which the participant had to deduce. The participant was asked to choose from multiple pictures which one best fitted the missing picture in the array. A sum score of correctly solved problems was calculated and added to the analyses as a control variable. The SPM test was administered on an iPad and lasted between 15 and 25 min.

#### Urbanization

A measure of urbanization was used to assess the population density in the areas where participants lived. This measure was based on the postal code that participants provided and using data from the Central Agency for Statistics, a Dutch governmental institution, a categorical variable was created that indicated the density of home addresses per postal code (ranging from 1 to 5: higher numbers indicating more addresses, so higher urbanization, per km^2^) (43). The measure was added to the analyses as a control variable. The question about the postal code was administered in a demographics questionnaire on the iPad.

#### Socioeconomic Status (SES)

A measure of SES was calculated based on the average yearly gross income per income receiver per household, separated per postal code areas. Postal codes were provided by the participants and information on the average yearly gross income was provided by the Central Agency for Statistics (43). The measure was added to the analyses as a control variable.

### Statistical Analysis

For the statistical analyses, multi-level models were used to allow for the nested structure of the data that implies dependency between the observations within the school classes. First, the overlap between autistic-like experiences and psychotic-like experiences was tested by regressing the DISC-C score on the ASSERT score, and we added a possible moderation by sex. A random intercept for class was added to allow for the nested structure of the data. Second, multi-level regression analyses were performed to investigate the effect of autistic-like experiences, psychotic-like experiences and the co-occurrence of both on the social cognition measures and the friendship measures. Two modeling procedures were created. Each of these procedures were repeated for the RMET, the two measures of the Dot perspective task, the three measures of the trust game and the two friendship measures. In all models, the social cognition measures and friendship measures served as the dependent variable. A first modeling procedure was created to examine the relationship between the dependent variable and the co-occurrence of autistic-like experiences and psychotic-like experiences. This was done by adding an interaction between the ASSERT score and the DISC-C score as predictor. A random intercept for class was added to allow for the nested structure of the data. A second modeling procedure was created to separately examine the relationship between the dependent variable and having autistic-like experiences or positive psychotic-like experiences. This was done by adding a main effect the ASSERT score and a main effect of the DISC-C score and a random intercept for class. Additionally, sex, the SPM score, the urbanization measure and the SES measure were added as control variables but removed in the final model when the predictors were not significant. Results of the final model of all modeling procedures are reported (and so, only when control variables were significant, they are reported). All models were fitted using the full maximum likelihood estimation method. Analyses were done in R version 3.5.1 using the R package “nlme” (44).

## Results

### Descriptives

First, we tested whether autistic-like experiences as measured with the ASSERT and positive psychotic-like experiences as measured with the DISC-C are related. The ASSERT score significantly predicted the DISC-C score [*t*_(289)_ = 3.2, *p* < 0.01]. See Table 1 for descriptive statistics per questionnaire and task.

**Table 1:** Descriptive statistics (mean and standard deviation) per questionnaire and task.

**Questionnaire/task**	**Mean score**
	**(standard deviation)**
DISC-C	0.8 (0.87)
ASSERT	3.92 (2.17)
RMET	18.1 (2.7)
Dot perspective task altercentric intrusion rate	1.05 (0.15)
Dot perspective task egocentric intrusion rate	1.07 (0.17)
Baseline trust behavior	3.9 (2.1)
Average trust behavior in the trustworthy condition	5.9 (3.01)
Average trust behavior in the untrustworthy condition	4.34 (2.93)
Indegree friendship	6.94 (2.92)
Outdegree friendship	8.05 (3.82)
SPM	44.51 (6.3)

### RMET Task

The RMET was used to test for mental state recognition skills. First, we tested the relationship between the RMET and the co-occurrence of autistic-like experiences and psychotic-like experiences. Results indicated no significant interaction effect of autistic-like experiences and psychotic-like experiences on the RMET score [*t*_(272)_ = −0.23, *p* = 0.82]. Second, we separately tested the relationship between the RMET and having autistic-like experiences or psychotic-like experiences. Results indicated no significant main effect of autistic-like experiences on the RMET score [*t*_(273)_ = −1.31, *p* = 0.19]. Results also showed no significant main effect of psychotic-like experiences on the RMET score [*t*_(273)_ = −1.51, *p* = 0.13]. A significant main effect of sex on the RMET score was found [*t*_(273)_ = −3.02, *p* < 0.01] with girls scoring higher compared to boys. A significant main effect of urbanization was found [*t*_(273)_ = 2.09, *p* < 0.05] with participants living in a more densely populated area scoring higher compared to participants living in a less densely populated area.

### Dot Perspective Task

The Dot perspective task measures perspective taking skills. The altercentric intrusion rate indicates the extent to which the avatar's perspective interfered with the participant's judgement when the participants had to adopt their own perspective. We first tested the relationship between the altercentric intrusion rate and the co-occurrence of autistic-like experiences and psychotic-like experiences. Results indicated no significant interaction effect of autistic-like experiences and psychotic-like experiences on the altercentric intrusion rate [*t*_(287)_ = 1.39, *p* = 0.17]. Second, we separately tested the relationship between the altercentric intrusion rate and having autistic-like experiences or psychotic-like experiences. Results indicate no effect of autistic-like experiences on the altercentric intrusion rate [*t*_(288)_ = −0.55, *p* = 0.59] and no effect of psychotic-like experiences on the altercentric intrusion rate [*t*_(288)_ = −0.03, *p* = 0.97]. The second measure we examined was called the egocentric intrusion rate and indicated the extent to which the participant's perspective interfered with their judgement when the participant needed to adopt the avatar's perspective. Results showed no significant interaction effect of autistic-like experiences and psychotic-like experiences on the egocentric intrusion rate [*t*_(287)_ = −1.17, *p* = 0.24]. Also, there was no significant main effect of autistic-like experiences on the egocentric intrusion rate [*t*_(288)_ = 0.3, *p* = 0.76] and no significant main effect of psychotic-like experiences on the egocentric intrusion rate [*t*_(288)_ = −0.56, *p* = 0.57].

### Trust Game

Baseline trust was calculated as the mean of the first trial investment in the trustworthy condition of the trust game and the first trial investment in the untrustworthy condition of the trust game. We first tested the relationship between baseline trust and the co-occurrence of autistic-like experiences and psychotic-like experiences. Results indicated no significant interaction effect of autistic-like experiences and psychotic-like experiences on baseline trust behavior [*t*_(286)_ = −0.82, *p* = 0.41]. We then tested the separate effects of having autistic-like experiences or psychotic-like experiences on baseline trust. Neither a significant effect of autistic-like experiences on baseline trust behavior [*t*_(287)_ = 0.76, *p* = 0.45] nor a significant effect of psychotic-like experiences on baseline trust behavior was found [*t*_(287)_ = −0.72, *p* = 0.47]. Results did show a sex difference in baseline trust with boys scoring higher compared to girls [*t*_(287)_ = 3.23, *p* < 0.01].

Average trust in the trustworthy condition of the trust game was indicated by the average investment of all trials in the trustworthy condition. Results indicated no significant interaction effect of autistic-like experiences and psychotic-like experiences on the average trust behavior in the trustworthy condition [*t*_(286)_ = 0.61, *p* = 0.54]. Also, no significant main effect of autistic-like experiences on average trust in the trustworthy condition was found [*t*_(287)_ = 1.52, *p* = 0.13]. The main effect of psychotic-like experiences on average trust behavior in the trustworthy condition was also not significant [*t*_(287)_ = 0.56, *p* = 0.58]. A main effect of sex on average trust behavior in the trustworthy condition was found with boys scoring higher than girls [*t*_(287)_ = 3.3, *p* < 0.01].

The average trust in the untrustworthy condition of the trust game was based on the average investment of all trials in the untrustworthy condition. No significant interaction effect of autistic-like experiences and psychotic-like experiences on the average trust behavior in the untrustworthy condition was found [*t*_(286)_ = −0.64, *p* = 0.52]. Furthermore, results did not indicate a significant main effect of autistic-like experiences on average trust in the untrustworthy condition [*t*_(287)_ = 0.19, *p* = 0.85] and no significant main effect of psychotic-like experiences on the average trust behavior in the untrustworthy condition [*t*_(287)_ = 1.11, *p* = 0.27]. Results did show a sex difference in average trust in the untrustworthy condition with boys scoring higher compared to girls [*t*_(287)_ = 3.71, *p* < 0.001].

### Friendship Measures

The outdegree measure of friendship indicated the number of pupils a participant selects as friends. We first examined the relationship between the outdegree measure and the co-occurrence of autistic-like experiences and psychotic-like experiences. Results indicated no significant interaction effect of autistic-like experiences and psychotic-like experiences on the outdegree score [*t*_(287)_ = −1.02, *p* = 0.21]. Furthermore, we separately tested the relationship between the outdegree measure and having autistic-like experiences or psychotic-like experiences. Results indicated no significant main effect of autistic-like experiences on the outdegree score [*t*_(288)_ = −1.24, *p* = 0.21] and no significant main effect of psychotic-like experiences on the outdegree score [*t*_(288)_ = 1.55, *p* = 0.12].

**Figure 1:**
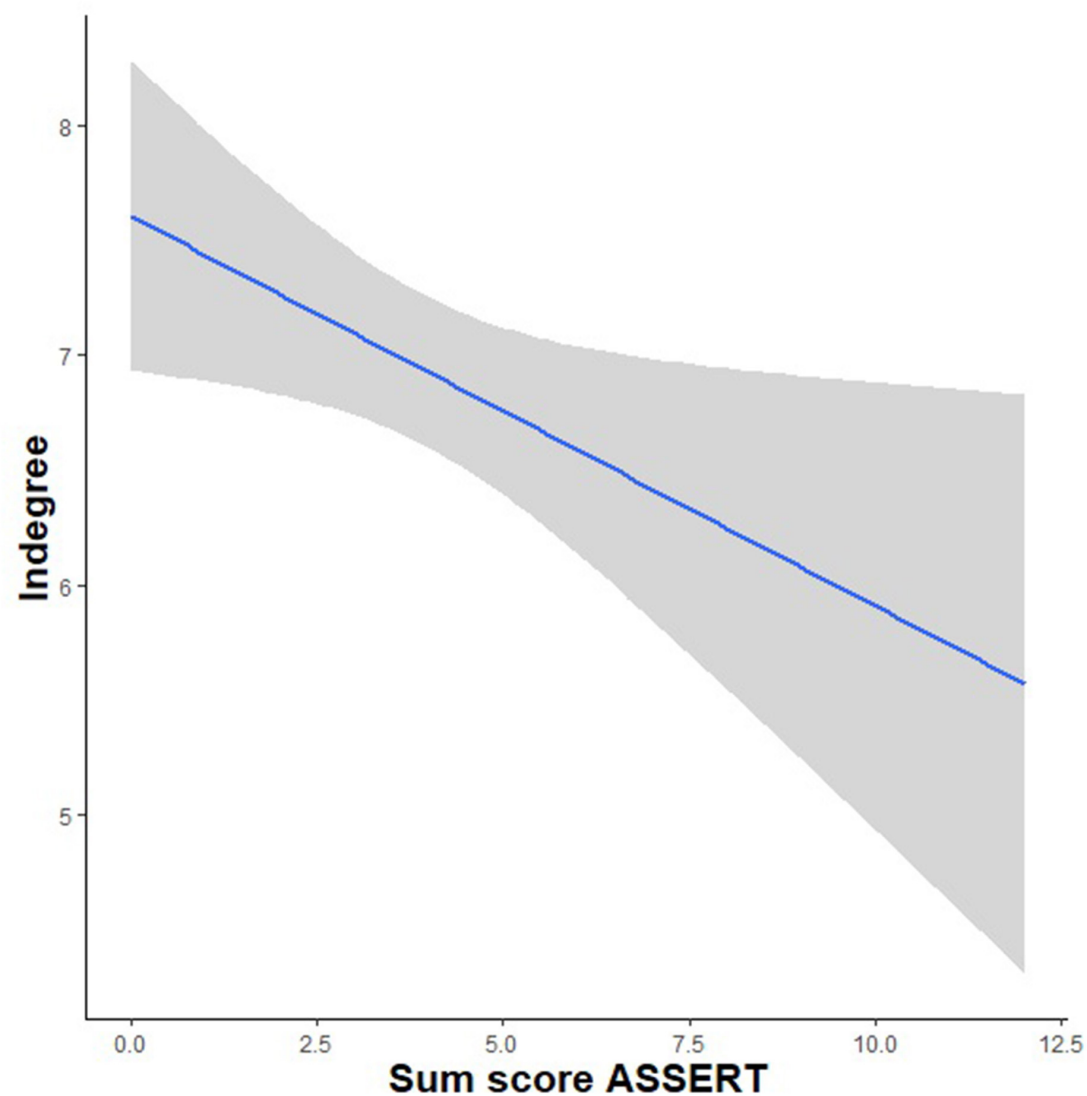
A significant, negative relationship between autistic-like experiences and the indegree friendship measure.

The indegree measure indicated the number of pupils selecting a participant as friend. Results showed no significant interaction effect of autistic-like experiences and psychotic-like experiences on the indegree score [*t*_(287)_ = 0.2, *p* = 0.84]. Also, results showed no significant effect of psychotic-like experiences on the indegree score [*t*_(288)_ = 0.21, *p* = 0.83]. Results did show a significant, negative effect of autistic-like experiences on the indegree score [*t*_(288)_ = −2.51, *p* = 0.01]. This means that those participants who showed higher overall levels of autistic-like experiences were selected as a friend less often by their classmates (see Figure 1), but there is no evidence that they themselves select fewer friends in their class.

## Discussion

In the current study, we examined performance on a comprehensive battery of social cognition tasks in a non-clinical sample of young adolescents with autistic-like experiences, positive psychotic-like experiences and with the co-occurrence of both. Secondly, we examined the effect of autistic-like experiences, psychotic-like experiences and their co-occurrence on friendships in daily life. Results showed a significant, positive relationship between autistic-like experiences and psychotic-like experiences. However, the current study did not find evidence for an association between autistic-like experiences, psychotic-like experiences, or the co-occurrence of both and the performance on any of the social cognition tasks. While psychotic-like experiences showed no significant positive or negative relationship with real-life friendships, social relationships were affected by autistic-like experiences. Specifically, we found a negative relationship between self-reported autistic-like experiences and the indegree measure of friendship, i.e., the number of classmates that selected the adolescent as their friend. Interestingly, we did not find evidence for this relationship with the outdegree measure of friendship, which reflects the number of classmates the adolescent themselves nominated as their friends.

### Social Cognition and Autistic-Like Experiences and Psychotic-Like Experiences

Both autistic-like experiences and positive psychotic-like experiences were reported by the participants, confirming previous research that these difficulties can also be found in samples that are considered to be typically developing. With regards to autistic-like experiences, the levels in our sample (*M* = 3.92, *sd* = 2.17) were slightly higher than those previously reported using the same instrument (35), though significantly below the suggested cut-off score of 8 for clinical diagnosis. While levels of reported psychotic-like experiences were low (*M* = 0.8, *sd* = 0.87), the prevalence was similar to earlier work using the same instrument in an early adolescent sample (34), as well as studies using other measures which have estimated the prevalence of psychotic symptoms at 17% in this age range (45).

In line with earlier studies in non-clinical groups, autistic-like experiences and psychotic-like experiences were significantly and positively associated in our sample (25, 26, 46–50). The result of the current study and of previous studies may indicate shared etiological mechanisms that drive increases in both autistic-like experiences and psychotic-like experiences due to genetic and environmental risk factors (2, 25). We hypothesized that this overlap would also be reflected in social-cognitive abilities but our results did not reveal any significant relationships between social cognition and autistic-like experiences, psychotic-like experiences or the combination of the two. The results of the current study underscore the probable shared etiology, however, in the current sample no altered functioning was found on a comprehensive battery of social cognition tasks in relation to either autistic- or psychotic-like experiences, so we cannot conclude how social cognition contributes to this shared etiology. Furthermore, we found a relationship between autistic-like experiences and social behavior (in terms of friendships) while no evidence for such an association was found for psychotic-like experiences. This shows the importance to not only look at the overlap and differences between ASC and SSC in terms of social cognition but also examine social behavior using daily life indicators. Specifically, friendship data provided by adolescents and also by their peers may shed light on alterations in social behavior that are not evident on the lab-based performance measures.

As described, previous studies in non-clinical samples of autism and psychosis did find that autistic-like experiences or psychotic-like experiences at a subclinical level are related to decreased ToM, emotion recognition and social skills (16–24). Several methodological factors may contribute to this discrepancy. For example, one of the measures we used was the trust game, which has not been used in previous studies examining subclinical experiences of ASC and SSC. Furthermore, the RMET is subject to debate on the exact social-cognitive processes that are involved (51). Another explanation for the differences in results could be the age of the sample as prior work was conducted in late adolescent and adult samples while the adolescents in the current study were around age 12. At this age, the foundational aspects of social cognition, such as following and interpreting the movements and gaze of others, and understanding their basic intentions are well-developed (52), but the higher-level aspects of social cognition, as well as the associated networks in the brain, continue to develop throughout adolescence and into adulthood (8, 53). These include the more complex forms of intentionality and ToM (54, 55), the ability to understand mixed and complex emotions (56, 57), and to flexibly adapt trust behavior based on social information (58). As we did not find evidence for an association between subclinical experiences and altered complex forms of social cognition, we hypothetically suggest that altered complex forms of social cognition in relation to these subclinical experiences may only be expressed during late adolescence or early adulthood but future research is needed to confirm this. In addition to these developmental considerations, it is important to note that the participants in our study were enrolled in the higher tracks of the Dutch educational system. The Dutch educational system has three main educational tracks based on academic performance. Schools from the lower track did not participate in the current study. Therefore, the enrolled participants had a relatively high level of education and cognitive performance, factors which are positively related to social cognition abilities and may have offset the potentially deleterious effects of autistic-like experiences and psychotic-like experiences (59).

The current sample provided the opportunity to test the off-cited diametric model of (subclinical) autism and psychosis, which predicts that the co-occurrence of ASC and SSC symptoms have an ameliorating effect on one another toward normality (2, 28). The study by Abu-Akel et al. (27) found support for the diametric model, as the performance on a social cognition task of people with both high autistic tendencies and high psychosis proneness was similar to that of people with low autistic tendencies and low psychosis proneness. As we did not find evidence for an association between performance on the social cognition tasks and subclinical experiences of autism or subclinical experiences of psychosis, we consequently also did not find evidence for a relationship between the co-occurrence of autistic-like experiences and psychotic-like experiences and the performance on the social cognition tasks, and thus did not find support for this model in our early adolescent sample. More studies are required to better understand the effect of the co-occurrence of both subclinical experiences to improve distinction and diagnoses.

### Friendships and Autistic-Like Experiences and Psychotic-Like Experiences

The current results showed that the more autistic-like experiences someone has, the less often the adolescent is selected as a friend by their peers. This may suggest that peers experience altered social behavior in young adolescents with autistic-like experiences while we did not find evidence that those adolescents themselves experience altered friendship behavior. Using peers as informants about the social behavior of adolescents with subclinical expressions of ASC or SSC has not often been done in prior work but the current study implicates peers can be a useful source of information. The current study also did not find evidence for an association between autistic-like experiences and the performance on computerized tasks tapping into social cognition. This implies that the computerized social cognition tasks do not tap into the mechanisms that make that the adolescents with autistic-like experiences are less often nominated as friends by their peers. If replicated, future studies could set out to further investigate these mechanisms, for example by using social cognition tasks with higher ecological validity or measured in the context of daily life using ecological momentary assessment and by using other informants (e.g., teachers, parents) to assess the social networks. Furthermore, we did not find evidence for an association between psychotic-like experiences and the two measures of friendship used, suggesting a possible difference in the social behavior in daily life of young adolescents with autistic-like experiences and young adolescents with psychotic-like experiences.

### Limitations and Future Directions

In the present study we used self-reported measures of autistic-like experiences and positive psychotic-like experiences. This requires the ability to assess and reflect on own behavior, which may have differed among the young adolescents in our sample. It should be noted, however, that these self-report measures are validated questionnaires also for this age group (33, 35). Further research using multiple informants (teacher, parents) would allow potential differences in assessment of subclinical experiences to be examined in more detail. In addition, future studies could use a measure of schizotypal traits rather than psychotic-like experiences. The current study was based on a homogenous sample in terms of age and level of education. This may limit the generalization of the results. At the same time, this homogeneity has likely reduced the differences between participants in the ability to reflect on their autistic-like and psychotic-like experiences and their social network ties. Due to the general design of this longitudinal study, some measures were administered at a 6-month interval. Furthermore, the peer nomination questionnaire was administered within classrooms. Using well-defined groups is a prerequisite for measuring (the dynamics of) complete social networks. However, this also implies that friendship ties outside the classroom could not be taken into account. Consequently, our measures will underestimate the total number of friends for most of the adolescents in our sample. However, as young adolescents spend a large amount of their time at school and all classes are taken with the same classmates, this social environment provides reliable information about a large part of their social interactions. Future research may add information about egocentric networks, as well as examine qualitative aspects of peer relationships.

### Conclusions

The current study did not find evidence for a relationship between autistic-like and/or positive psychotic-like experiences and social-cognitive functioning in young adolescents. Furthermore, there was no evidence for a relationship between psychotic-like experiences and friendships. In contrast, having autistic-like experiences was negatively related to the number of times being selected as a friend by peers even though there was no evidence for a lower number of self-reported friends. This study provides initial evidence that information provided by peers may shed light on (altered) social behavior associated with autistic-like experiences that is not apparent on performance measures, as well as elucidate possible differences between autistic- and psychotic-like experiences.

## Data Availability Statement

The raw data supporting the conclusions of this article will be made available by the authors, without undue reservation.

## Ethics Statement

The studies involving human participants were reviewed and approved by Vaste Commissie voor Wetenschap en Ethiek, Faculty of Behavioral and Movement Sciences, Vrije Universiteit Amsterdam (VCWE). Written informed consent to participate in this study was provided by the participants' legal guardian/next of kin.

## Author Contributions

LK, NL, and HS developed the main conceptual idea. HS, MH, and RW collected the data. HS and BB developed the analytical models and carried out all analyses. HS wrote the initial draft of the paper. NL, MH, RW, BB, MB, and LK provided feedback on the draft and approved the final version. All authors contributed to the article and approved the submitted version.

## Conflict of Interest

The authors declare that the research was conducted in the absence of any commercial or financial relationships that could be construed as a potential conflict of interest.
